# Exploring Ventilator-Associated Pneumonia: Microbial Clues and Biomarker Insights from a Retrospective Study

**DOI:** 10.3390/medicina60081346

**Published:** 2024-08-19

**Authors:** Ahmed M. Gouda, Ashraf E. Sileem, Hanan M. Elnahas, Ahmed E. Tawfik, Refaat A. Eid, Ayed A. Shati, Saleh M. Al-Qahtani, Samy A. Dawood, Mohammed A. Alshehri, Mohamed Eissa, Mohamed A. Soltan, Ahmed E. Noreldin, Amir Helmy Elwishahy, Essamedin M. Negm

**Affiliations:** 1 Department of Pharmacy Practice, Faculty of Pharmacy, Zagazig University, Zagazig 44519, Egypt; 2 Department of Chest Diseases, Faculty of Medicine, Zagazig University, Zagazig 44519, Egypt; 3 Department of Pharmaceutical and Industrial Pharmacy, Faculty of Pharmacy, Zagazig University, Zagazig 44519, Egypt; 4 Zagazig University Hospitals, Zagazig 44519, Egypt; 5 Pathology Department, College of Medicine, King Khalid University, Abha 62529, Saudi Arabia; 6 Department of Child Health, College of Medicine, King Khalid University, Abha 62529, Saudi Arabia; 7 Clinical Pathology Department, Faculty of Medicine, Zagazig University, Zagazig 44519, Egypt; 8 Department of Microbiology and Immunology, Faculty of Pharmacy, Sinai University, Ismailia 41611, Egypt; 9 Department of Histology and Cytology, Faculty of Veterinary Medicine, Damanhour University, Damanhour 22511, Egypt; 10 General and Onco Surgery Department, Dhahran Aljanoub General Hospital, Aseer 7363, Saudi Arabia; 11 Department of Anaesthesia, ICU and Pain Management, Faculty of Medicine, Zagazig University, Zagazig 44519, Egypt

**Keywords:** ventilator-associated pneumonia (VAP), broncho alveolar lavage (BAL), Sequential Organ Failure Assessment (SOFA), Acute Physiology and Chronic Health Evaluation II (APACHE II) scores

## Abstract

*Background and Objectives*: Ventilator-associated pneumonia (VAP) is a common complication in critically ill patients receiving mechanical ventilation. The incidence rates of VAP vary, and it poses significant challenges due to microbial resistance and the potential for adverse outcomes. This study aims to explore the microbial profile of VAP and evaluate the utility of biomarkers and illness severity scores in predicting survival. *Materials and Methods*: A retrospective cohort study was conducted involving 130 patients diagnosed with VAP. Microbial analysis of bronchoalveolar lavage (BAL) fluid, as well as measurements of C-reactive protein (CRP) and procalcitonin (PCT) levels, were performed. Sequential Organ Failure Assessment (SOFA) and Acute Physiology and Chronic Health Evaluation II (APACHE II) scores were calculated to assess illness severity. Statistical analyses were conducted to determine correlations and associations. *Results*: The study revealed that *Klebsiella pneumoniae* (*K. pneumoniae*) (50.7%) and *Pseudomonas aeruginosa* (*P. aeruginosa*) (27.69%) were the most identified microorganisms in VAP cases. SOFA (*p*-value < 0.0001) and APACHE II (*p*-value < 0.0001) scores were effective in assessing the severity of illness and predicting mortality in VAP patients. Additionally, our investigation highlighted the prognostic potential of CRP levels (odds ratio [OR]: 0.980, 95% confidence interval [CI] 0.968 to 0.992, *p* = 0.001). Elevated levels of CRP were associated with reduced survival probabilities in VAP patients. Conclusion: This study highlights the microbial profile of VAP and the importance of biomarkers and illness severity scores in predicting survival. *Conclusions*: The findings emphasize the need for appropriate management strategies to combat microbial resistance and improve outcomes in VAP patients.

## 1. Introduction

Ventilator-associated pneumonia (VAP) is a type of lung infection that develops in patients who are on mechanical ventilation, which is a life-support intervention used in critical care settings. VAP is considered to be a nosocomial or healthcare-associated infection [1]. It frequently occurs as a complication in patients who are critically ill, particularly manifesting after a period of at least 48 h of being subjected to mechanical ventilation [2]. The incidence rates of VAP can vary depending on factors such as the population studied, healthcare settings, and regional differences, typically ranging from 8% to 28% among mechanically ventilated patients in intensive care units [3]. VAP is a significant concern in hospitals as it can lead to longer hospital stays, increased healthcare costs, and even life-threatening situations [4].

One of the challenges in managing VAP is the issue of microbial resistance. The bacteria causing VAP are often resistant to multiple antibiotics, making treatment difficult and contributing to worse outcomes for patients [5]. The overuse and misuse of antibiotics greatly contribute to the emergence of antibiotic-resistant bacteria [6]. Clinicians must use antibiotics judiciously and take measures to prevent resistance. Delayed initiation of antibiotic therapy and inadequate treatment can lead to unfavorable outcomes, including the emergence of multidrug-resistant (MDR) pathogens [7].

To diagnose VAP, Broncho alveolar lavage (BAL) is commonly used to obtain fluid samples from the lower respiratory tract [8]. BAL helps identify the specific bacteria or pathogens causing the infection and their susceptibility to antibiotics. This information guides the selection of appropriate antibiotics and helps prevent the development of antibiotic resistance [9]. In addition to BAL, biomarkers such as C-reactive protein (CRP) and procalcitonin (PCT) levels are measured in blood samples to aid in the diagnosis and monitoring of VAP. Elevated CRP and PCT levels can indicate the presence of infection and help assess the response to treatment [10].

To ascertain the severity of the illness and forecast the probability of mortality among critically ill patients, including those with VAP, scoring systems such as the Sequential Organ Failure Assessment (SOFA) and Acute Physiology and Chronic Health Evaluation II (APACHE II) are valuable tools [11]. These scores consider various physiological parameters and laboratory values to provide a quantitative measure of the patient’s overall health status. Monitoring changes in these scores over time can provide insights into treatment response and the risk of complications [12].

The overarching objective of this study was to provide insights into the microbial etiology, clinical characteristics, and prognostic factors for VAP using a retrospective cohort design.

## 2. Materials and Methods

### 2.1. Ethical Approval and Informed Consent

The study protocol was approved by the Institutional Review Board (IRB) under code 9998/19-10-2022. Informed consent was obtained from all patient’s legal representatives prior to their inclusion in the study.

### 2.2. Study Design

**Figure 1:**
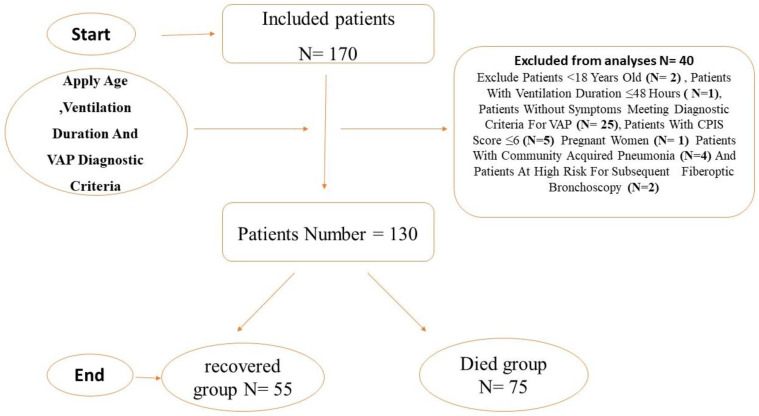
Study flow diagram.

We conducted a retrospective cohort study to investigate the microbial profile of BAL fluid in patients with VAP and emphasized the significance of utilizing SOFA and APACHE II scores as reliable tools for assessing illness severity and predicting mortality. Furthermore, we aimed to investigate the relationships between inflammatory biomarkers (CRP, PCT), disease severity scores (SOFA, APACHE II), age, gender, and survival in patients with VAP. The study flow diagram is shown in (Figure 1).

### 2.3. Participants

A total of 130 patients diagnosed with VAP between November 2022 and June 2023 were included in the study. These patients were admitted to the Emergency, Surgical, and Respiratory Intensive Care Units in Zagazig University Hospitals. All patients diagnosed with VAP during the study period were enrolled and followed for outcomes. Survival was censored at the time of hospital discharge. The participants were subsequently categorized into two groups based on their outcomes: the “died” group, consisting of 75 patients who did not survive until hospital discharge, and the “recovered” group, consisting of 55 patients who survived until hospital discharge.

### 2.4. Inclusion Criteria

Adult patients aged 18 years or older who underwent invasive mechanical ventilation for a duration exceeding 48 h and exhibited symptoms meeting the diagnostic criteria for Ventilator-Associated Pneumonia (VAP). The diagnosis of VAP was determined using the Clinical Pulmonary Infection Score (CPIS), as proposed by Luna et al. in 2003 [13]. A CPIS score of more than 6 was considered indicative of VAP.

### 2.5. Exclusion Criteria

Patients with community-acquired pneumonia and pregnant women were excluded from this study. Patients who were deemed to be at high risk for a subsequent fiberoptic bronchoscopy (patients with bleeding diathesis, recent acute myocardial infarction, seizures, raised intracranial pressure, and those on very high respiratory support) were excluded.

### 2.6. Data Collection and Procedures

1. Patients who fulfilled the criteria for inclusion in this study underwent the following procedures to determine the presence of VAP: a thorough medical history was taken from the relative(s) of the patients, a full clinical examination consisting of both general and local chest examinations was performed, and a plain chest X-ray was performed using a portable PRACTix 33 Pluse Philips (Rome, Italy) X-ray machine. The pulmonary infiltration seen on the chest X-ray served as a diagnostic indicator for the diagnosis of VAP;
2. Laboratory investigations in the form of the following tests were performed. These included the measurement of complete blood count including white blood cell count, red blood cell count, hemoglobin level, and platelet count. C-reactive protein levels were measured using a Ruche Cobas800-702 auto analyzer manufactured by Ruche Diagnostic in Germany. Procalcitonin levels were measured using the BioMerieux’s VIDAS BRAHMS PCT assay manufactured by Biomerieux in Durham, NC, USA;
3. Empirical antibiotic therapy was initiated according to the IDSA (Infectious Diseases Society of America) 2016 guidelines once the criteria for suspected VAP were met. This means that when there were indications that a patient might have VAP based on specific clinical criteria, antibiotic treatment was started without waiting for the confirmation of the causative organism through laboratory tests. The choice of antibiotics was guided by the guidelines provided by the IDSA in 2016, which outline recommendations for the treatment of infectious diseases [14];
4. The SOFA score was calculated on admission for all included patients, which was based on clinical information and lab results for predicting ICU mortality;

The SOFA score was calculated based on six organ systems: respiratory, cardiovascular, hepatic, coagulation, renal, and neurological. Each system was evaluated based on clinical and laboratory parameters, and the highest score for each system was added together to give a total SOFA score. The scale of SOFA scores encompasses a range from 0 to 24, wherein higher scores denote more pronounced levels of organ dysfunction severity. SOFA scores could be useful in predicting mortality and assessing the severity of illness in critically ill patients. However, it is important to interpret SOFA scores in the context of other clinical factors and patient-specific characteristics.

5. APACHE II is a severity of illness classification system that is commonly used in critical care settings. The score was calculated based on 12 physiological variables and age, and ranges from 0 to 71, with higher scores indicating greater severity of illness. The 12 physiological variables included vital signs, blood gas measurements, electrolyte levels, and other laboratory values. Each variable was assigned a score based on the degree of abnormality, and the scores were added together to give the total APACHE II score. APACHE II scores could provide valuable prognostic information and guide clinical decision-making in critically ill patients;
6. Fiberoptic bronchoscopy with BAL under aseptic conditions was performed.

The timing of Broncho alveolar lavage (BAL) action was determined based on the presence of VAP criteria. This meant that the decision to perform BAL, which involved the washing of the airways to collect fluid samples, was made once the specific criteria indicative of VAP were met. The timing of performing BAL was determined based on the fulfillment of these criteria [15].

After administering 3.5–7.5 mg of IV midazolam as sedation, oxygen at 100% was given and ventilator settings were adjusted. The bronchoscope was wedged into the segment bronchus likely involved based on imaging and at least 100 mL of specimen was obtained after instilling 150 mL of sterile saline. The specimen was sent immediately for culture, with >1% squamous cells indicating contamination.

7. Microbiological analysis for the BAL: The sample was inoculated on blood agar, chocolate agar (incubated aerobically and anaerobically at 37 °C), and McConkey agar (incubated aerobically at 37 °C). Additionally, a sample was inoculated into a brain–heart infusion broth. The colony-forming units for growth-positive plates were determined [16].

### 2.7. Statistical Analysis

All data are expressed as the mean ± SD for numeric variables and as the number (percentage) for categorical variables. Comparisons of numerical variables were determined by unpaired *t*-tests. Comparisons of categorical variables were assessed by a χ^2^ (Chi-square). A *p*-value < 0.05 was considered to indicate statistical significance. Pearson correlation coefficients between APACHE II and SOFA scores and each of the laboratory values were calculated. A multivariate logistic regression analysis was performed to assess the relationship between the measured parameters and VAP survival. Data were analyzed using Graph Pad Prism version 8.2 and SPSS version 26.

## 3. Results

### 3.1. Demography and Baseline Characteristic

Table 1 provides information on patient characteristics of those who died and those who recovered from VAP. The mean age of those who died was 58 years, which was significantly higher than the mean age of those who recovered (46 years). There were more male patients in both sets, but the difference was not statistically significant. Patients who died had a shorter time before mechanical ventilation (mean 1.52 days) compared to those who recovered (mean 5.21 days), and they were mechanically ventilated for a longer period (mean 17.52 days) than those who recovered (mean 9.36 days). The duration of the ICU stay did not show any substantial disparity between the two groups. Most chest X-rays showed bilateral involvement in both groups. The most common reasons for ICU admission were a disturbed consciousness level and septic shock.

### 3.2. Diversity of Microbial Profiles across VAP Patient Diagnosis

As shown in Table 2, the diversity of microbial profiles among VAP patients varied significantly across different diagnoses. *P. aeruginosa* was notably prevalent in cases of burst abdomen and diabetic foot, scoring 9 and 8, respectively. MRSA emerged as the dominant pathogen in RTA (Road Traffic Accident) cases, scoring 5. Klebsiella exhibited notable prevalence in various diagnoses including RTA, AECOPD (Acute Exacerbation of Chronic Obstructive Pulmonary Disease), diabetic foot, brain tumor, burst abdomen, cervical fracture, and ILD (Interstitial Lung Disease). *E. coli* was commonly found in asthma, burst abdomen, diabetic foot, ILD, and OSA (Obstructive Sleep Apnea) cases, each scoring 1, while Acinetobacter predominated in cases of LL Ischemia, scoring 4. These findings underscore the importance of understanding microbial profiles in different clinical contexts among VAP patients.

### 3.3. Microbiological Profile of VAP Patients

**Figure 2:**
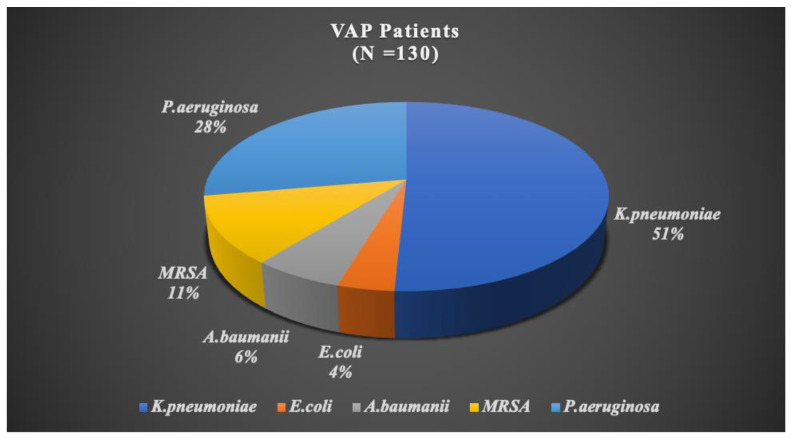
A pie chart representing the microbiological profile of VAP Patients.

In this study, we investigated the microbiological profile of VAP patients (N = 130). The distribution of microorganisms revealed that *K. pneumoniae* was the most prevalent, accounting for 50.7% of cases, followed by *P*. *aeruginosa* at 27.69%. Additionally, Methicillin-resistant *Staphylococcus aureus* (MRSA) was identified in 11.5% of cases, *Acinetobacter baumanii (A. baumanii)* in 6.1%, and *Escherichia coli (E. coli)* in 3.84%. These findings highlight the prominent role of *K. pneumoniae* and *P*. *aeruginosa* as the most identified microorganisms in VAP patients in our study (Figure 2).

### 3.4. White Blood Cells, Platelet Count, C-Reactive Protein, Procalcitonin, SOFA, and APACHE II Scores in Died and Recovered Groups of VAP Patients

**Figure 3:**
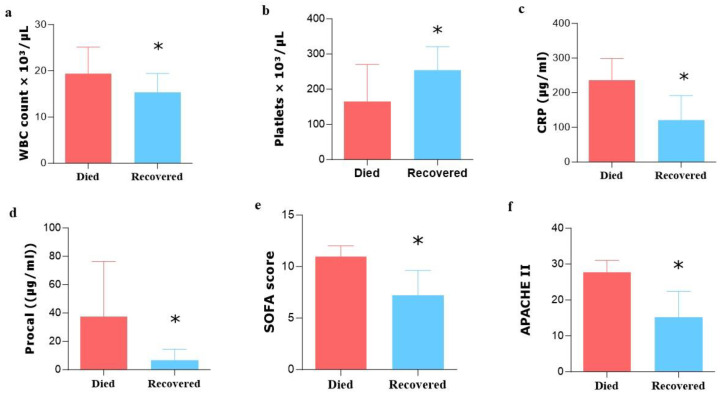
Bar charts showing the comparative scores between the “died” and “recovered” groups of VAP Patients. (**a**) WBC count, (**b**) platelet count, (**c**) CRP, (**d**) procalcitonin, (**e**) SOFA, and (**f**) APACHE II Scores. * *p*-value < 0.001.

In comparison to recovered patients, those who passed away showed significantly higher levels of white blood cells (WBCs), CRP, PCT, SOFA, and APACHE II scores. However, patients who recovered had notably higher platelet counts compared to those who did not survive (Figure 3).

### 3.5. Analysis of Antibiotic Susceptibility Patterns in Frequently Isolated Pathogens

**Figure 4:**
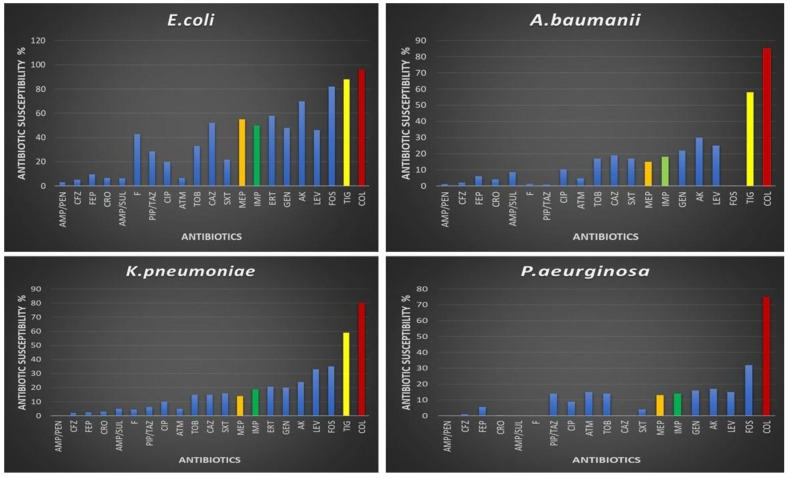
Antibiotic susceptibility profiles of *E. coli*, *A. baumanii*, *K. pneumoniae*, and *P. aeruginosa.* Abbreviations: AMP/PEN (ampicillin/penicillin), CFZ (cefazolin), FEP (cefepime), CRO (ceftriaxone), AMP/SUL (ampicillin/sulbactam), F (nitrofurantoin), PIP/TAZ (piperacillin/tazobactam), CIP (ciprofloxacin), ATM (aztreonam), TOB (tobramycin), CAZ (ceftazidime), SXT (sulfa-trimethoprim), MEP (meropenem), IMP (imipenem), ERT (ertapenem), GEN (gentamycin), AK (amikacin), LEV (levofloxacin), FOS (Fosfomycin), TIG (tigecycline), COL (colistin).

**Figure 5:**
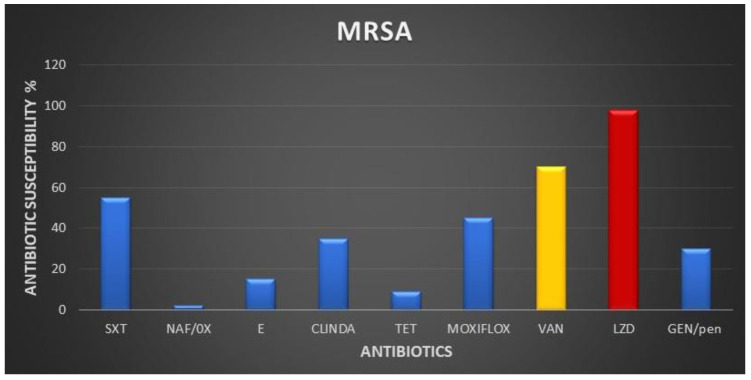
Antibiotic susceptibility of Gram-positive MRSA. Abbreviations: NAF/OX (nafcillin/oxacillin), E (erythromycin), CLINDA (clindamycin), SXT (sulfa trimethoprim), TET (tetracycline), GEN/pen (gentamycin/penicillin), MOXIFLOX (moxifloxacin), VAN (vancomycin), LZD (linezolid).

Our study revealed significant variability in the antibiotic susceptibility profiles of the most commonly isolated Gram-negative pathogens. Notably, colistin demonstrated the highest effectiveness, exhibiting sensitivity rates of 96% for *E. coli*, 85% for *A. baumannii*, 80% for *K. pneumoniae*, and 75% for *P*. *aeruginosa*. Tigecycline also exhibited favorable sensitivity rates, with 88% for *E. coli*, 58% for *Acinetobacter*, and 59% for *K. pneumoniae*. However, carbapenem sensitivity rates were notably low, with only 19% and 14% sensitivity rates observed for imipenem and meropenem in *K. pneumoniae*, 50% imipenem and 55% meropenem sensitivity rates in *E. coli*, 18% imipenem and 15% meropenem sensitivity rates in *A. baumannii*, and 14% imipenem and 13% meropenem sensitivity rates in *P. aeruginosa* (Figure 4). Furthermore, Gram-positive MRSA exhibited a 70% sensitivity to vancomycin, while demonstrating an impressive 98% sensitivity to linezolid (Figure 5).

### 3.6. The Correlations between SOFA and APACHE II Scores and Laboratory Values

**Figure 6:**
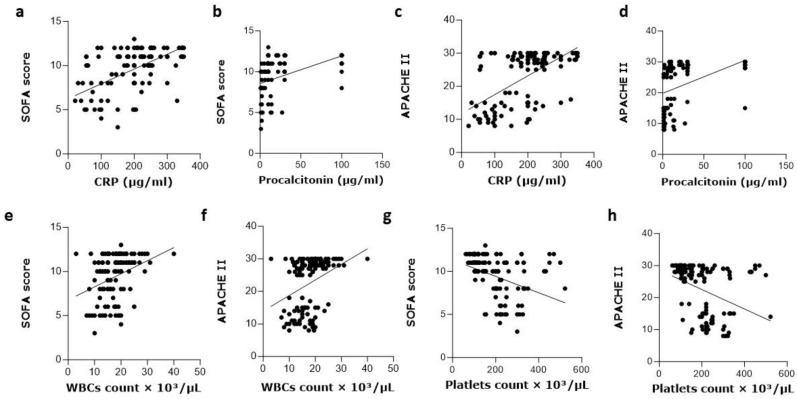
Correlations between APACHE II and SOFA scores and multiple laboratory values in VAP patients. (**a**,**b**) correlation between SOFA score and CRP and Procalcitonin. (**c**,**d**) correlation between APACHE II and CRP and Procalcitonin. (**e**,**f**) correlation of SOFA score and APACHE II with WBCs count, respectively. (**g**,**h**) correlation of SOFA score and APACHE II with platelets count, respectively.

We conducted a Pearson correlation analysis to assess the relationships between APACHE II and SOFA scores and various laboratory values (WBCs, platelets, CRP, and PCT) among VAP patients in our study sample. The correlation analysis showed noteworthy positive relationships between SOFA score and CRP (r^2^ = 0.3317, *p* < 0.0001) and PCT (r^2^ = 0.1975, *p* < 0.0001), indicating that higher levels of CRP and PCT were linked to more severe illness according to the SOFA score (Figure 6a,b). Similarly, a significant positive correlation was found between APACHE II score and CRP (r^2^ = 0.3691, *p* < 0.0001) and PCT (r^2^ = 0.1920, *p* < 0.0001), suggesting that higher CRP and PCT levels were associated with greater disease severity according to the APACHE II score (Figure 6c,d). Additionally, a positive correlation was observed between WBC count and both SOFA (r^2^ = 0.1016, *p* = 0.0002) and APACHE II scores (r^2^ = 0.1025, *p* = 0.0002), indicating a potentially more severe systemic inflammatory response in critically ill patients (Figure 6e,f). Furthermore, a significant negative correlation was found between platelet counts and both SOFA (r^2^ = −0.1422, *p* < 0.0001) and APACHE II scores (r^2^ = −0.1404, *p* < 0.0001), suggesting that higher platelet counts were associated with less severe illness according to both scoring systems (Figure 6g,h). These findings provide important insights into the relationships between disease severity scores and laboratory values in VAP patients, highlighting the potential value of these scores in assessing critical illness.

### 3.7. Logistic Regression Analysis of the Measured Parameters in Relation to VAP Survival

A multivariate logistic regression analysis was performed to assess the relationship between the measured parameters and VAP survival. The analysis included CRP, PCT, SOFA score, APACHE II score, age, and gender as independent variables, and survival to hospital discharge as the dependent variable. The findings from the regression analysis indicated that CRP (OR: 0.980, 95% CI 0.968 to 0.992, *p* = 0.001) was a significant predictor of survival in VAP patients. PCT, SOFA, APACHE II, and age were not significant predictors. The probability of recovery was six times higher in males compared to females (Table 3).

## 4. Discussion

The current study examined clinical features, microbiological profiles, illness severity, and inflammatory biomarkers in VAP cases to determine what factors are linked to mortality outcomes. We compared data from patients who died and recovered from VAP to gain an understanding of survival predictors that could assist with clinical management and prognosis forecasting.

Important findings on patient characteristics provide insights into elements that may influence outcomes in VAP cases. We noticed that advanced age was connected to an increased risk of death in VAP situations. This aligns with earlier research that showed worse results in older patients with critical diseases. Advanced age is usually accompanied by frailty, malnutrition, decreased organ reserve, and higher occurrence of comorbid conditions, which increase susceptibility and impair response to infection [17]. We did not find a significant difference in mortality between males and females. However, larger studies are needed to determine if gender influences VAP outcomes [18].

The findings of this study revealed significant variability in the microbial profiles of Ventilator-Associated Pneumonia (VAP) patients across different clinical diagnoses, underscoring the complexity and heterogeneity of VAP influenced by underlying conditions. Consistent with previous research, the data showed that *P. aeruginosa* was notably prevalent in patients with burst abdomen and diabetic foot, likely due to compromised immune responses and open wounds in these conditions. Studies have similarly reported a high prevalence of *P. aeruginosa* in patients with surgical wounds and diabetic foot ulcers due to the pathogen’s ability to thrive in moist environments and its resistance to multiple antibiotics [19]. However, a study by Craven et al. (2009) suggested that the prevalence of *P. aeruginosa* might be overestimated in some clinical settings due to sample contamination and selection biases in patient populations [20]. MRSA emerged as the dominant pathogen in Road Traffic Accident (RTA) cases, linked to extensive soft tissue injuries and frequent use of invasive devices, which aligns with earlier studies highlighting MRSA’s prevalence in trauma patients. For example, research by Stone et al. (2012) found a high incidence of MRSA infections in trauma patients due to invasive procedures and prolonged hospital stays [21]. Contrarily, a study by Craxford et al. (2021) indicated a declining trend in MRSA prevalence among trauma patients due to improved infection control practices and the increased use of decolonization protocols [22]. *K. pneumoniae* species were widespread across various diagnoses, reflecting its adaptability and resistance mechanisms, a pattern corroborated by literature emphasizing *K. pneumoniae*‘s significant presence in hospital settings due to its capability to form biofilms and resist antibiotics. Studies have noted that *Klebsiella pneumoniae* can colonize various body sites and is associated with severe infections in immunocompromised patients, highlighting its significant role in hospital-acquired infections [23]. However, other research, such as that by Gupta et al. [24], points out that while *K. pneumoniae* remains prevalent, its dominance is being challenged by emerging resistant strains of *Enterobacteriaceae*, which could shift the current understanding of pathogen prevalence in hospital settings.

*E. coli*, while less frequent, was found in asthma, burst abdomen, diabetic foot, ILD, and Obstructive Sleep Apnea (OSA) cases. Although less common in VAP, *E. coli*’s presence in these conditions necessitates vigilance, especially in immunocompromised patients or those with prolonged hospital stays. Research has indicated that *E. coli*, though typically associated with urinary tract infections, can also be a significant pathogen in VAP, particularly in patients with weakened immune systems [25]. Conversely, a study by Ma et al. [26] suggested that *E. coli* is often a secondary pathogen in VAP cases, overshadowed by more virulent bacteria such as *P. aeruginosa* and *K. pneumoniae*, questioning its primary role in VAP infections. *A. baumannii* species predominated in Lower Limb (LL) Ischemia cases, highlighting its notorious ability to survive in hospital environments and its resistance to multiple antibiotics, consistent with findings from other studies focused on critically ill patients. *A. baumannii* has been recognized in several studies for its resilience and ability to cause outbreaks in intensive care units, particularly among patients with extensive tissue damage or prolonged ventilation needs. In contrast, research by Dijkshoorn et al. [27] emphasized that while *A. baumannii* is highly resilient, its prevalence varies significantly across different geographical regions and hospital settings, suggesting that local epidemiological factors play a crucial role in its distribution. These diverse microbial profiles suggest the need for tailored antibiotic therapy based on prevalent pathogens in specific conditions to enhance treatment efficacy and reduce antibiotic resistance. For instance, the high prevalence of *P. aeruginosa* in burst abdomen and diabetic foot cases necessitates antipseudomonal coverage, while the dominance of MRSA in RTA patients requires anti-MRSA agents. The widespread occurrence of *K. pneumoniae* indicates a need for its inclusion in empirical therapy for VAP. Understanding these specific microbial landscapes is essential for developing targeted antimicrobial strategies, improving patient outcomes, and combating antibiotic resistance. Future research should focus on longitudinal studies to monitor changes in microbial profiles and resistance patterns, and on rapid diagnostic tools for timely and precise pathogen identification in VAP patients.

The most common organisms isolated in our study were Gram-negative bacilli such as *K. pneumoniae* and *P. aeruginosa*, as well as Gram-positive bacteria such as MRSA. Multidrug-resistant organisms were also identified, highlighting the challenge in treating VAP [28]. Patients who died were on mechanical ventilation for a longer duration and required ventilation earlier in their clinical course. Prolonged mechanical ventilation is a known risk factor for VAP and is associated with worse outcomes [29].

The majority of patients in both groups had bilateral involvement on chest imaging, suggesting advanced lung disease at presentation. Septic shock and decreased consciousness, the most common reasons for ICU admission, are severe manifestations of critical illness and were likely significant contributors to mortality.

Elevated levels of WBCs, CRP, and PCT in the deceased group indicate an exaggerated immune response associated with severe infections. These biomarkers commonly assess infection severity and systemic inflammation. Higher levels in deceased patients suggest an uncontrolled inflammatory response, contributing to organ dysfunction and a fatal outcome [30]. A study on VAP patients yielded similar results, linking higher WBCs, CRP, and PCT levels to increased mortality. These biomarkers serve as valuable prognostic indicators in VAP patients [31].

The significantly elevated SOFA scores observed in patients who did not survive, along with higher APACHE2 scores, underscore the severity of organ impairment and its impact on patient physiology [32]. The results of our investigation support the findings that indicated a steady connection between elevated SOFA and APACHE2 scores and heightened mortality rates in critically ill patients [33]. These findings collectively support the notion that SOFA and APACHE2 scores can reliably predict mortality in patients with VAP.

Interestingly, a contrasting platelet count pattern was noted between the two sets of VAP patients. Recovered patients had significantly higher platelet counts compared to non-survivors. Platelets play a crucial role in the immune response and blood clot formation. The lower platelet counts in the deceased group may indicate coagulation system dysregulation, leading to an increased risk of bleeding complications or disseminated intravascular coagulation (DIC). On the other hand, higher platelet counts in the recovered group suggest a more effective immune response and better infection control. Our study findings are consistent with recent research that showed a clear association between low platelet counts and adverse outcomes in critically ill patients, including higher mortality rates. This suggests that monitoring platelet count could serve as a straightforward and valuable prognostic marker for poor outcomes in critically ill individuals with infectious complications, such as VAP [34].

The antibiotic sensitivity profiles observed among the major Gram-negative bacteria isolated in our study, including *E. coli, A. baumannii, K. pneumoniae,* and *P. aeruginosa*, demonstrated significant variability. Colistin exhibited the highest sensitivity rates of all antibiotics tested, with 96%, 85%, 80%, and 75% effectiveness against *E. coli, A. baumannii, K. pneumoniae,* and *P. aeruginosa*, respectively. These findings correlate well with previous work that also reported colistin as one of the most reliably active agents against multidrug-resistant Gram-negative pathogens [35]. In contrast, our current results showed notably low sensitivity to carbapenems across all organisms: *E. coli* demonstrated only 50% sensitivity to imipenem, while sensitivity was 45% or less for *A. baumannii, K. pneumoniae*, and *P. aeruginosa*. These observations support the concerns raised by Meletis in 2016 regarding the increasing resistance of these Gram-negative bacteria species to carbapenems [36]. Given the limited efficacy of carbapenems observed in our study, as well as reports of emerging resistance from other researchers, there is a clear need to develop alternative treatment strategies and novel antimicrobials that can effectively combat these multidrug-resistant Gram-negative infections [37]. Continuous surveillance of local resistance patterns also remains important for optimizing empiric therapy and treatment guidance.

The study confirmed a vancomycin sensitivity of approximately 70% among MRSA isolates, aligning with previous research [38]. Despite some resistance, vancomycin remains effective against the majority of MRSA strains globally, supporting its continued frontline use. Additionally, linezolid demonstrated a high success rate of 98% against MRSA, suggesting its potential as an important treatment alternative. These findings underscore the importance of exploring effective alternatives such as linezolid, particularly when traditional antibiotics may be less effective, to improve patient outcomes and combat antibiotic resistance.

Correlation analysis demonstrated significant positive associations between SOFA score and both CRP and PCT. Similarly, the APACHE II score showed significant positive correlations with CRP and PCT, indicating that elevated levels of CRP and PCT are linked to more severe illness as reflected by higher SOFA and APACHE II scores. These results are consistent with previous studies by S. Wang and Chen in 2015, which also reported similar positive correlations between inflammatory markers and disease severity in critically ill patients [39]. Additionally, statistical analysis showed a positive correlation between WBC count and both SOFA score and APACHE II score, suggesting a heightened systemic inflammatory response and greater disease severity in critically ill patients with elevated WBC counts [40]. A significant negative correlation between platelet counts and both SOFA score and APACHE II score was found, indicating that higher platelet counts are linked to lower SOFA and APACHE II scores, implying less severe illness. These results are in line with a published study, which reported similar inverse correlations between platelet counts and disease severity in critically ill patients [41]. However, it is important to note that our study focused specifically on patients with VAP, and further research is warranted to validate these findings in larger and more diverse patient cohorts. Additionally, future studies should explore the predictive value of these correlations in guiding clinical decision-making and prognostication.

In the current study, several factors predicting outcomes in VAP patients were identified. Specifically, a significant association between CRP levels and survival was observed, with higher CRP levels indicating a lower probability of survival, consistent with previous research on CRP as a prognostic marker in VAP. [42]. CRP, an acute phase protein, rises in response to infection and inflammation, likely reflecting a more severe inflammatory response to pulmonary infection, leading to poorer clinical outcomes. However, markers such as PCT, SOFA score, and APACHE II score did not emerge as significant predictors of survival in our cohort, possibly due to differences in patient characteristics, treatment regimens, and sample size compared to other studies, highlighting the need for larger studies to validate these findings. Interestingly, males exhibited a higher probability of survival compared to females, consistent with prior findings regarding gender disparities in critical illness outcomes. [43]. This discrepancy may be influenced by biological and immunological differences, as well as variations in comorbidities and treatments received. Further investigation into the role of gender in VAP outcomes would be valuable.

While VAP has been extensively studied, our research offers several unique contributions that enhance its novelty. Firstly, it focuses on the microbial profile in our specific region, revealing the prevalence of *Klebsiella pneumoniae* and *Pseudomonas aeruginosa*. This regional focus helps tailor local treatment guidelines and antibiotic use. Secondly, we combined the use of CRP, PCT levels, SOFA, and APACHE II scores to predict patient outcomes, offering a thorough assessment of VAP prognosis. Lastly, our study highlights the importance of early and appropriate management strategies to combat microbial resistance in ICU settings. By using biomarkers and severity scores, our findings provide useful insights for clinicians to improve patient care.

There are a few limitations to our study. It was conducted retrospectively at a single center and involved a relatively small number of participants. Additionally, we did not control for other comorbidities, which could have influenced the outcomes. The linear methods used may not fully capture non-linear relationships, suggesting that future studies should incorporate non-linear models to provide a more comprehensive understanding of biomarkers and severity scores in predicting VAP outcomes. To validate our results and better understand the effectiveness of different biomarkers and severity scores in predicting outcomes for VAP patients, more comprehensive studies are required.

## 5. Conclusions

In critically ill VAP patients, our study found *K. pneumoniae* and *P. aeruginosa* as the most prevalent microorganisms isolated from BAL fluid. Additionally, SOFA and APACHE II scores emerged as reliable tools for assessing illness severity and predicting mortality. Furthermore, Our findings also indicated that higher CRP levels were associated with reduced survival probabilities, although the presence of inflammatory comorbidities may have influenced these levels. Therefore, while CRP and other biomarkers can provide valuable insights into the inflammatory status of patients, they should be interpreted with caution and in conjunction with other clinical factors. Implementing early identification, appropriate treatment, and the judicious use of biomarkers and severity scores may enhance outcomes in critically ill patients with VAP. However, further research is needed to validate these findings and to explore the potential role of these biomarkers in guiding clinical decision-making, including the duration and intensity of antibiotic therapy.

## Figures and Tables

**Table 1:** Patients’ characteristics in died and recovered groups of VAP patients.

	Died N (%)	Recovered N (%)	*p*-Value
Age (mean ± SD)	58 ± 13.5	46 ± 16.7	<0.0001
Gender (M/F)	43 (52)/32 (68)	40 (48)/15 (32)	0.096 (NS)
**Days before MV** (mean ± SD)	1.52 ± 6.1	5.21 ± 4.4	0.0002
**Ventilation Days** (mean ± SD)	17.52 ± 6.3	9.36 ± 3.2	<0.0001
**Length of ICU stay** (mean ± SD)	19.27 ± 10.22	18.45 ± 9.07	0.63 (NS)
**Chest X-ray**			
Right	7	25	<0.0001
Left	6	14	
Bilateral	62	16	
**Comorbidities**			
AECOPD	2	13	
ASTHMA	0	2	
Brain abscess	1	1	
Brain tumor	6	0	
Burst abdomen	15	3	
Cervical Fracture	6	0	
colon cancer	1	0	
Diabetic foot	15	4	
DVT	0	1	
Hydrocephalus	2	1	
ICH	1	1	
ILD	1	5	
LL Ischemia	6	0	
metastasis	1	0	
OSA	0	1	
PE	1	5	
RTA	12	16	
SAH	4	2	
Stroke	1	0	
**Indication of ICU admission ** **(Admission diagnosis)**			
altered level of consciousness	27 (20.7%)	21 (16.1%)	<0.0001
Septic shock	39 (30%)	8 (6.1%)	
Spinal shock	5 (3.8%)	0 (0%)	
Type I RF	2 (1.5%)	9 (6.9%)	
Type II RF	2 (1.5%)	17 (13%)	

Values are represented as mean ± SD or n (%). Abbreviations: CRP, C-reactive protein; PCT, Procalcitonin; SOFA, Sequential Organ Failure Assessment; APACHE II, Acute Physiology and Chronic Health Evaluation II; AECOPD, Acute exacerbation of chronic obstructive pulmonary disease; DVT, deep vein thrombosis; ICH, Intracerebral Hemorrhage; ILD, Interstitial Lung Disease; OSA, Obstructive Sleep Apnea; PE, Pulmonary Embolism; RTA, Road Traffic Accident; SAH, Subarachnoid Hemorrhage. NS, non significant.

**Table 2:** Diversity of microbial profiles across VAP patient diagnosis.

Diagnosis	Pseudomonas	MRSA	Klebsiella	*E. coli*	Acinetobacter	Total
AECOPD	5	1	8		1	15
ASTHMA	1			1		2
Brain abscess			2			2
Brain tumor	2		4			6
Burst abdomen	9	2	6	1		18
Cervical Fracture			6			6
colon cancer	1					1
Diabetic foot	8	2	8	1		19
DVT			1			1
Hydrocephalus	1	1	1			3
ICH			2			2
ILD			5	1		6
LL Ischemia	1	1			4	6
Metastasis	1					1
OSA				1		1
PE	2	1	2		1	6
RTA	4	5	18		1	28
SAH	1	2	3			6
Stroke					1	1
Total	36	15	66	5	8	130

**Table 3:** Logistic regression analysis of the measured parameters in relation to VAP survival.

	Estimate	*p*-Value	OR
CRP	−0.020	0.001	0.980
PCT	−0.16	0.423	0.984
SOFA score	−0.544	0.202	0.581
APACHE II	−0.180	0.122	0.835
Age	0.014	0.627	1.014
Gender	1.887	0.028	6.600

OR odds ratio.

## Data Availability

The datasets generated and analyzed during the current study are not publicly available but are available from the corresponding author upon a reasonable request.
